# Prognostic value of maximum standard uptake value, metabolic tumor volume, and total lesion glycolysis of positron emission tomography/computed tomography in patients with breast cancer: A systematic review and meta-analysis

**DOI:** 10.1371/journal.pone.0225959

**Published:** 2019-12-11

**Authors:** Weibo Wen, Dongchun Xuan, Yulai Hu, Xiangdan Li, Lan Liu, Dongyuan Xu

**Affiliations:** 1 Department of Nuclear Medicine, Affiliated hospital of Yanbian University, Yanji, Jilin Province, China; 2 Center of Morphological Experiment, Medical College of Yanbian University, Yanji, Jilin Province, China; 3 Department of Pathology, Affiliated hospital of Yanbian University, Yanji, Jilin Province, China; Ente Ospedaliero Cantonale, SWITZERLAND

## Abstract

**Purpose:**

A comprehensive systematic review of the literature was conducted on parameters from ^18^ F-FDG PET and a meta-analysis of the prognostic value of the maximal standard uptake value (SUVmax), metabolic tumor volume (MTV) and total lesional glycolysis (TLG) in patients with breast cancer (BC).

**Patients and methods:**

Relevant English articles from PubMed, EMBASE, and the Cochrane Library were retrieved. Pooled hazard ratios (HRs) were used to assess the prognostic value of SUVmax, MTV, and TLG.

**Results:**

A total of 20 primary studies with 3115 patients with BC were included. The combined HRs (95% confidence interval [CI] of higher SUVmax and higher TLG for event-free survival (EFS) were 1.53 (95% CI, 1.25–1.89, *P* = 0.0006) and 5.94 (95% CI, 2.57–13.71, *P* = 0.97), respectively. Regarding the overall survival (OS), the combined HRs were 1.22 (95%CI, 1.02–1.45, *P* = 0.0006) with higher SUVmax, and 2.91(95% CI, 1.75–4.85, *P* = 0.44) with higher MTV. Higher MTV showed no correlation with EFS [1.31(95% CI, 0.65–2.65, *P* = 0.18)] and similarly higher TLG showed no correlation with OS [1.20(95% CI, 0.65–2.23, *P* = 0.45)]. Subgroup analysis showed that SUVmax, with a median value of 5.55 was considered as a significant risk factor for both EFS and OS in BC patients.

**Conclusion:**

Despite clinically heterogeneous BC patients and adoption of various methods between studies, the present meta-analysis results confirmed that patients with high SUVmax are at high risk of adverse events or death in BC patients, high MTV predicted a high risk of death and high TLG predicted a high risk of adverse events.

## Introduction

Breast cancer (BC) is the most common malignancy in women. Although new imaging tools and assisted systemic therapy have improved the survival rate of patients with BC, patients with early invasive BC are still at risk of recurrence or death. It is crucial to identify patients experiencing a risk of relapse or progression, as there is no clinical method for accurate assessment of the prognosis and survival of BC patients till date.

According to the latest report, tumor size, nuclear grade, axillary lymph node involvement, hormone receptor (e.g., estrogen receptor (ER) progesterone) status receptor (PR) and human epidermal growth factor receptor 2 (HER2), and ki-67 proliferation index might act as effective factors in predicting the recurrence or progression in patients at high risk.[1] A growing body of evidence suggests that fluoro18-fluorodeoxyglucose (^18^F- FDG) positron emission tomography (PET/CT) has a great prognostic significance in predicting malignant tumors, TNM staging, evaluation of therapeutic effects, FDG parameter SUV Max, metabolic tumor volume (MTV) and total lesional glycolysis (TLG), as a parameter of tumor metabolism and volume have also received more and more attention. MTV is the size of the tumor tissue, which actively ingests ^18^F- FDG, and TLG is the median SUV value in the region of interest MTV [2–5].

However, it is still controversial whether the parameters of ^18^F-FDG PET/CT predict the survival rate of BC patients. Some studies reported significant relationships between high SUV max and poor prognoses in patients with BC[6–8], whereas no such correlation is observed by Alexandre Cochet et al. [9]. Therefore, a meta-analysis was designed to evaluate the prognostic value of SUV max MTV and TLG in BC patients.

## Material and methods

### Registration

This systematic review and meta-analysis was reported by following the guidelines of preferred reporting items of the systematic review and meta-analysis (PRISMA) statement[10].

### Inclusion criteria and literature source retrieval strategy

A systematic search of PubMed, Embase, and Cochrane Library (2012-May 2019) using the following keywords (“breast cancer” OR “breast carcinoma”)AND(“positron emission tomography” OR “positron emission tomography-computed tomography” OR “positron emission tomography computed tomography” OR “PET”OR “PET-CT” OR “PET CT” OR “PET/CT” OR“fluorodeoxyglucose” OR“FDG”) AND (“prognostic” OR “prognosis” OR “predictive” OR “survival” OR “outcome”) was performed. Inclusion criteria were as follows: (1) studies should include histologically diagnosed BC patients; (2) 18F-FDG PET/CT was used as imaging tool before treatment; (3) the study should at least report one form of survival data; and (4) articles published in English. The exclusion criteria were as follows: (1) studies that focused only on diagnosis, staging, or monitoring recurrence or progression; (2) studies involving patients with recurrent disease before treatment; and (3) reviews, case reports, conference abstracts and editorial materials. According to the inclusion and exclusion criteria, two authors independently conducted the search and screening, and any discrepancies were resolved by reaching a consensus. If the results reported are from the same sample, completed studies with the latest information will be used.

### Data extraction

Two authors (W Wen and D Xu) independently extracted the following data regarding the included studies (Table 1): (1) basic information of the study, including the year of publication, first author, study time, follow-up duration and study design; (2) details of patients and tumors, including median age, sample size, histology, TNM staging, treatment measures and endpoint. The information regarding 18F-FDG- PET scan data and parameters, determination of fasting time before injection, blood glucose detection before injection, determination of truncated interval value of FDG injection dose, extraction of truncated value of PET parameters SUV Max, MTV, TLG, and tumor profile was also extracted and presented in Table 2.

**Table 1:** Characteristics of included studies.

Study	Year	Country	Study period	Follow-up duration (months)	Median age (range), years	No.of patients	TNM staging	End points	study design	Histology	Treatment
Selin Carkaci et al.(2013)[11]	2013	USA	2005–2009	29(9–55)	56(33–80)	53	I-IV	OS	R	Inflammatory breast cancer	NAC
Sung Gwe Ahn et al.(2014)[12]	2014	Korea	2004–2008	6.23 year	48(25–80)	305	I-II	RFS	R	NA	CMT/ET
Jongtae Cha et al.(2018)[7]	2018	Korea	2008–2013	46.2(5.4–95.2)	50(27–82)	524	I-II	RFS	R	IDC/ILC/other	SG+NAC
Young Hwan Kim et al.(2015)[13]	2015	Korea	2010–2012	28.4(28.4±9.0)	50.5(30–76)	119	II/III	RFS	R	IDC	SG+CMT/ RT/ET
Seung Hyup Hyun et al.(2016)[14]	2016	Korea	2006–2012	39	46.1 ± 10.8	332	I-III	RFS	R	NA	SG/others
SUYUN CHEN etal.(2017)[15]	2017	China	2003–2011	71(8–118)	51	86	II/III	EFS OS	R	NA	NAC
Ji-hoon Jung et al.(2017)[16]	2017	Korea	2008–2010	46 (29–79)	48.1 (39–79)	131	I-III	PFS	R	IDC	SG+CMT/ RT/ET
Seung Hyun Son et al.(2014)[9]	2014	Korea	2007–2008	53.6(7–66)	NA	123	I-III	OS	R	IDC	SG/CMT/Hormone therapy
Kazuhiro Kitajima et al.(2017)[6]	2017	Japan	2012–2015	32.3	57.7 (30–90)	73	I-III	DFS	R	IDC/ILC/ others	SG+AC/RT/ET
Yannan Zhao et al.(2018)[17]	2018	China	2011–2015	NA	59 (37–78)	27	IV	PFS	R	NA(MBC)	500fulvestrant
Takayuki Kadoya et al.(2013)[18]	2013	Japan	2006–2011	52	58.0 ± 12.5	344	I-III	RFS	R	IDC/ILC others	SG
Jian Zhang et al.(2013)[19]	2013	China	2007–2010	26.6(14.–51.2)	52(18–70)	134	IV	PFS OS	P	NA(MBC)	CMT/RT/Hormone therapy
Ana María Garía Vicente et al.(2015)[20]	2015	Spain	2009–2013	34.8	54	198	I-III	DFS OS	R	IDC/ ILC/	SG+NAC
Alexandre Cochet et al.(2013)[9]	2013	France	2006–2010	30(9–59)	51(25–85)	142	II-IV	PFS	P	IDC/ ILC/ others	NA
Suyun Chen rt al.(2015)[21]	2015	China	2006–2011	65 (3–106)	51.9(23–87)	240	III-IV	PFS OS	R	IDC/ ILC/ others	SG/CMT/ET
Seung Hyun Son et al.(2015)[22]	2015	Korea	2006–2011	36.4(0.8–71.4)	49.1(29–75)	40	I-IV	OS	R	IDC	CMT/Hormone therapy
Mehdi Taghipour et al.(2015)[23]	2015	USA	2000–2012	28.5(0–94)	60 ± 14	78	I-IV	0S	R	Ductal carcinoma, Lobular carcinoma Others unknown	SG+CMT
Jahae Kim et al.(2012)[24]	2012	Korea	2006–2008	50 (17–73)	52 (32–83)	53	I-III	EFS OS	R	IDC/ILC/ others	SG+CMT/ RT/ET
Brett Marinelli et al.(2016)[25]	2016	USA	2001–2012	12.4	54±12	47	NA	OS	R	NA(MBC)	SG+CMT/ RT
Jang Yoo, et al.(2017)[26]	2017	Korea	2010–2014	30.9(6.6–61.8)	42.7 (29.5–51.8)	66	I-IV	RFS	R	IDC	SG+CMT/ RT

Abbreviations: NA = not available; R = retrospective; P = prospective; RFS = recurrence/relapse free survival; PFS = progression-free survival; DFS = disease-free survival; OS = overall survival; IDC = invasive ductal carcinoma; ILC = invasive lobular carcinoma; SG = surgery; CMT = chemotherapy; RT = radiotherapy; ET = endocrine therapy; NAC = neoadjuvant chemotherapy; AC = adjuvant chemotherapy.

**Table 2:** Methods of 18 F-FDG PET imaging of included studies.

Study	Duration of fasting	Preinjection blood glucose -test	Post-Injection interval	Dose of 18F-FDG	Pet parameters	Determina tion of cut- off values	Cut-off values		
							SUV	MTV(cm 3)	TLG
Selin Carkaci et al.(2013)[11]	6h	<150mg/dL	70±10 min	(555–629 MBq, 15–17 mCi)	SUV max	Others	3.8		
Sung Gwe Ahn et al.(2014)[12]	8h	<130mg/dL	60min	0.14 mCi/kg	SUV max	ROC curve	4		
Jongtae Cha et al.(2018)[7]	6h	<140mg/dL	60min	5.5MBq/kg	SUV max	ROC curve	6.75		
Young Hwan Kim et al.(2015)[13]	6h	150mg/dL	NA	3–5 MBq/kg	SUV max	ROC curve	11.1		
Seung Hyup Hyunet al.(2016)[14]	6h	200mg/dL	NA	5.0 MBq/kg	SUV max	others	7.0		
SUYUN CHEN et al.(2017)[15]	6h	200mg/dL	60min	259–555 MBq	SUV max	ROC curve	2.5		
Ji-hoon Jung et al.(2017)[16]	6h	150mg/dL	60min	4.8MBq/kg	SUV max	ROC curve	5.5		
Seung Hyun Son et al.(2014)[27]	6h	150mg/dL	60min	8.1MBq/kg	SUV max MTV TLG	ROC curve	5.6	8.55	14.43
Kazuhiro Kitajima et al.(2017)[6]	*5h*	160mg/dL	60min	4.0MBq/kg	SUV max MTV TLG	ROC curve	3.6	3.15	16.0
Yannan Zhao et al.(2018)[17]	6h	10 mmol/L	60min	7.4MBq/kg	SUV max MTV TLG	others	6.09	18.78	72.5
Takayuki Kadoya et al.(2013)[18]	4h	150mg/dL	60-90min	3.7MBq/kg	SUV max	ROC curve	3.0		
Jian Zhang et al.(2013)[19]	6h	7.8mmol/L	50-70min	7.4 MBq/kg	SUV max	ROC curve	3.54		
Ana María García Vicente et al.(2015)[20]	4h	160 mg/dL	60min	370 MBq	SUV max	ROC curve	6.05		
Alexandre Cochet et al.(2013)[9]	6h	NA	60min	5MBq/kg	SUV max	others	5.7		
Suyun Chen et al.(2015)[21]	6h	200mg/dL	60-90min	259–555 MBq	SUV max	others	6.0		
Seung Hyun Son et al.(2015)[22]	6h	150mg/dL	60min	8.1MBq/kg	SUV max	ROC curve	9.4		
Mehdi Taghipour et al.(2015)[23]	4h	200mg/dL	NA	5.55MBq/kg	SUV max MTV TLG	ROC curve	2.9	7.9	11.85
Jahae Kim et al.(2012)[24]	6h	8.3mmol/L	60min	7.4MBq/kg	SUV max MTV	others	7.3	11.1	
Brett Marinelli et al.(2016)[25]	NA	<200 mg/dL	60-90min	444–555 MBq	MTV TLG	others		NA	NA
Jang Yoo, et al.(2017)[26]	6h	140mg/dL	60min	5.18MBq/kg	TLG	others			52.38

Abbreviations: ROC = receiver operating characteristic, SUV max = maximum standard uptake value; MTV = metabolic tumor volume; TLG = total lesional glycolysis; NA = not available.

### Statistical analysis

We followed the same methodology as used in our previous study[28]. Event-free survival (EFS) is defined as the time from treatment initiation to recurrence or progression. In this meta-analysis, disease-free survival (DFS), progression-free survival (PFS), and disease-free metastasis survival in the included studies were combined and redefined as EFS. Overall survival (OS) was defined as the time from therapy initiation till death regardless of the causes[29, 30]. As the effect size of each study, hazard ratio (HR) and 95% confidence interval (CI) take into account the number and time of events, they are considered more accurate and reliable than odds ratio (OR) and relative risk (RR). HR and 95% CI were used to combine the data, and measure the effect of 18F-FDG PET parameters on survival outcome through effect size of HR in order to measure the correlation between SUV max, MTV and TLG values and the prognosis of BC patients. HR is the sum of differences between Kaplan-Meier survival curves and represents comparison of the two groups during a certain follow-up period. The impact of SUV max, MTV and TLG on survival was measured by HR. Data regarding multivariate HR and 95% CI were directly extracted from studies. If multivariate HR was not available, then univariate HR would be extracted. If both multivariate HR and univariate HR were unavailable, then the methodology recommended by Parmar et al[26] would be used to reconstruct HR estimate and its variance based on the survival data from Kaplan-Meier survival curves read by Engauge Digitizer (version 9.4). HR greater than 1 implies worse survival in patients with high SUV max, MTV or TLG, whereas HR less than 1 implies a survival benefit in patients with high SUV max, MTV or TLG.

Statistical heterogeneity was measured using chi-squared Q test and I^2^ statistic. Heterogeneity was considered to be present if *P*<0.05 or/and *I* ^2^ >50%. A fixed effects model was used for meta-analysis when heterogeneity was not significant, while a random effects model was used if heterogeneity was significant. RevMan version 5.3 (RevMan, version 5.3; The Nordic Cochrane Centre, The Cochrane Collaboration) and STATA version 12.0 (STATA Corp., College Station, TX) were used for statistical analysis. Begg’s test and Egger’s test were used for evaluating bias by STATA version 12.0. P values of less than 0.05 were considered to be statistically significant.

## Results

### Search results

**Fig 1:**
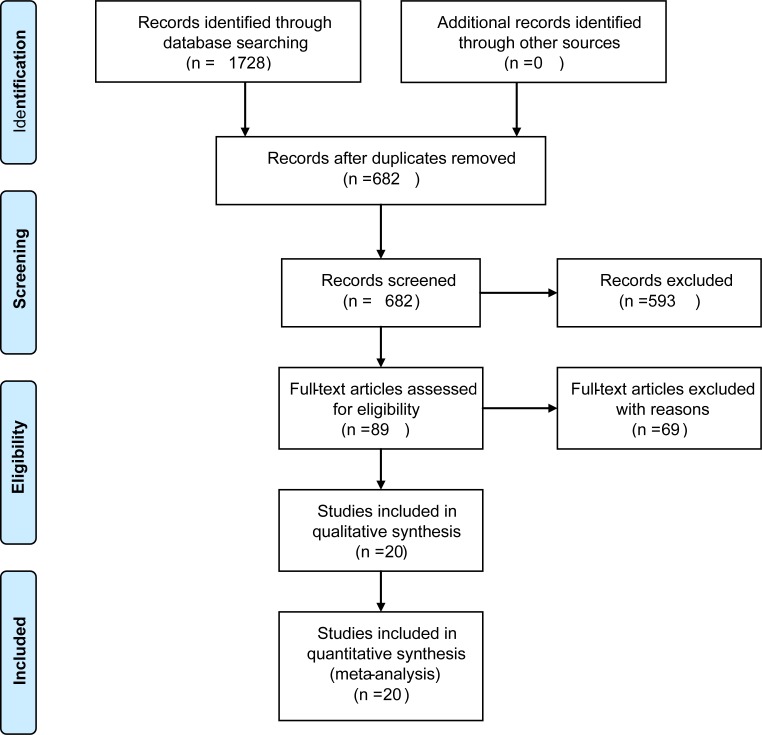
Flow diagram of study selection.

The search process of the literature was presented in (Fig 1). Search was conducted in three databases, which obtained 559 Embase articles, 1149 PubMed articles and 20 Cochrane Library articles initially (1728 articles). After excluding duplications and meeting summaries, 69 articles that did not meet the inclusion criteria were removed. Finally, 20 studies including 3,115 patients that met the conditions of the study and published from 2013 to 2019 were included in this meta-analysis[6, 7, 9, 11–27] (Fig 1). All 20 studies reported the prognostic values of SUVmax, MTV or TLG for BC survival.

### Literature quality evaluation was included

**Fig 2:**
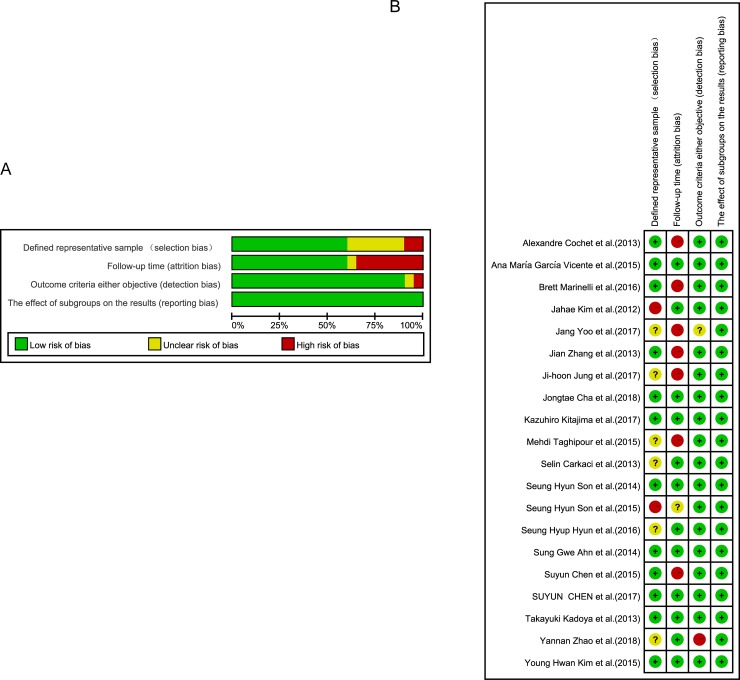
(a) Risk of bias graph: review authors’ judgments about each risk of bias item presented as percentages across all included studies. (b) Risk of bias summary: judgment of review authors regarding each risk of bias item for each included study.

The quality of 20 studies was assessed according to CRITICAL APPRAISAL OF PROGNOSTIC STUDIES (https://www.cebm.net/wp-content/uploads/2018/11/Prognosis.pdf), (Fig 2). Generally, the included studies were of high quality, In the domain of prognostic factor follow-up time measurements, there was a high risk of bias in 7 studies because follow-up data were missing Or the follow-up time was too short. 7 studies were judged to be at high or unclear risk of bias in the domain of defined representative sample measurements because few studies were non-blinded or non-randomized. Most of the studies were well described and monitored regarding adverse events by objective criteria.

### Study characteristics

Almost all the studies were conducted in Asia, with 9 in South Korea, 4 in China and 2 in Japan, 3 in the United States and 1 each in France and Spain. Two were prospective and 18 were retrospective studies. In the 18 SUVmax studies, the SUV cut-off values ranged from 2.5–11.1, which included 14 items with EFS as prognosis and 9 items with OS as prognosis. Among the 6 studies that measured MTV, 3 included EFS as prognosis and 4 included OS as prognosis. Among the 6 studies measuring TLG, 3 with EFS as prognosis and 3 with OS as prognosis were included. In addition, information such as age of the subjects during the follow-up period of tumor pathological staging was extracted. The details of all studies included in the analysis, histology and treatment included in all studies are presented in Table 1. Sixty-five percent of patients are with invasive ductal carcinoma (IDC), invasive lobular carcinoma (ILC), and other pathologies. One study [8] involved patients with inflammatory BC, and three studies[12, 17, 27] included only patients with advanced metastatic BC and all these included one or more treatments of surgery (SG)/chemotherapy (CMT)/radiotherapy (RT)/ endocrine therapy (ET)/ hormone /neoadjuvant chemotherapy (NAC). Yannan Zhao et al. 's study[17] involves experimental treatment of metastatic BC with fulvestrant.

### Primary outcome: EFS

**Fig 3:**
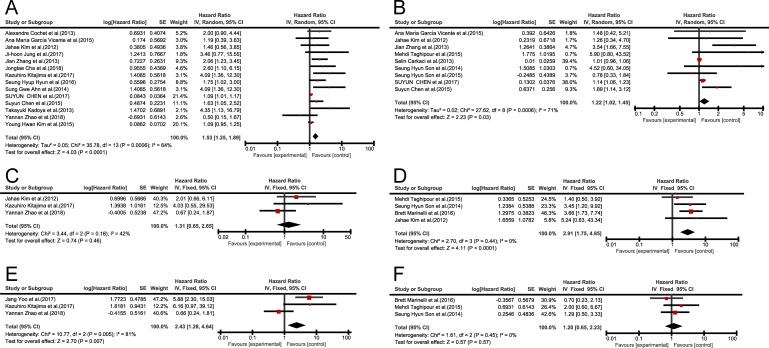
**Forest plots of HR for EFS and OS with SUVmax (A, EFS; B, OS), MTV (C, EFS; D, OS) and TLG (E, EFS;F, OS).** Chi-square test is a measurement of heterogeneity. P < 0.05 indicates significant heterogeneity. Squares = individual study point estimates. Horizontal lines = 95%CIs. Rhombus = summarized estimate and its 95%CI. Fixed: fixed effect model. Random: random effects model.

**Fig 4:**
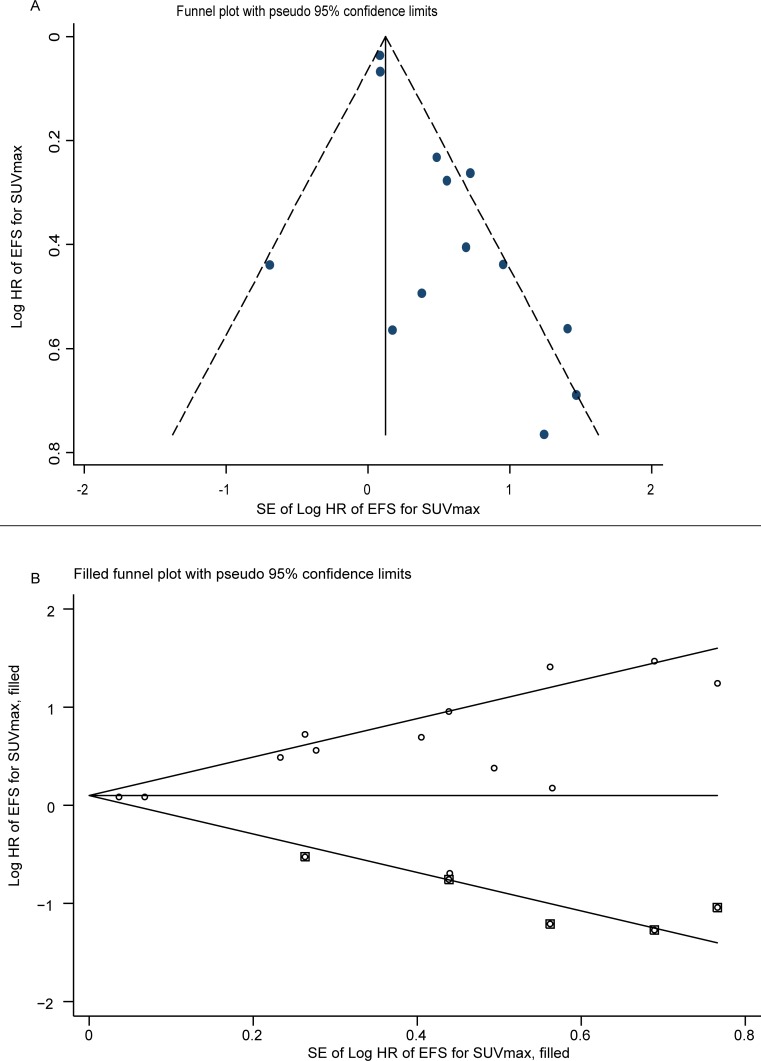
Funnel plots without (up column) and with (low column) trim and fill. The pseudo 95% confidence interval (CI) is computed as a part of analysis to produce funnel plots and their corresponding 95%CI for a given standard error (SE). HR indicates hazard ratio.

Fourteen studies analyzed EFS with SUVmax. After combining HR, the higher SUVmax, and the worse EFS are predicted. Fixed effects model (HR = 1.14; 95% CI = 1.07–1.21, *P* = 0.0006; *I*^*2*^ = 64%) showed statistical significance, and heterogeneity existed between studies, while random effects model [HR = 1.53; 95% CI = 1.25–1.89, *P* = 0.0006 (Fig 3A)] still showed meaningful results (Table 3). Potential publication bias was assessed by two statistical tests (Begg’s and Egger’s). Begg’s test showed no significant publication bias (*P* = 0.352), and Egger’s test (S1 Fig) indicated that there might be publication bias (*P* = 0.002). Therefore, trim and fill analysis was conducted to ensure the reliability of combined HR. Symmetrical funnel plots were obtained after trim and fill analysis (Fig 4). After adding the hypothesis literature, the results were obtained (HR = 1.104; 95% CI: 1.040–1.172), and no substantial change was observed in the results before and after adding the hypothesized literatures, which still showed that SUVmax was significantly correlated with EFS. Sensitivity analysis was conducted to further estimate the impact on combined HRs. Exclusion of each study showed no significant reduction in heterogeneity.

**Table 3:** Summary of meta-analysis results.

Endpoint	Metabolic parameter	No.of studies	Model used	HR	95% CI of HR	P value of HR	Heterogeneity I^2^ (%)	Conclusion
EFS	SUV max	14	Fixed effect	1.14	1.07–1.21	0.0006	64	significant
			Random effect	1.53	1.25–1.89	0.0006		significant
	MTV	3	Fixed effect	1.31	0.65–2.65	0.18	42	insignificant
	TLG	3	Fixed effect	2.43	1.28–4.64	0.005	81	significant
			Random effect	2.70	0.54–13.44	0.005		insignificant
OS	SUV max	9	Fixed effect	1.06	1.02–1.10	0.0006	71	significant
			Random effect	1.22	1.02–1.45	0.0006		significant
	MTV	4	Fixed effect	2.91	1.75–4.85	0.44	0	significant
	TLG	3	Fixed effect	1.20	0.65–2.23	0.45	0	insignificant

Abbreviations: HR = hazard ratios; CI = confidence interval, EFS = event-free survival ; OS = overall survival; SUV max = maximum standard uptake value; MTV = metabolic tumor volume; TLG = total lesional glycolysis.

Additional subgroup analyses were performed according to the cutoff method, threshold, analysis method and endpoint (Table 4). Among the studies that included EFS as endpoint, studies that adopted cutoff method using ROC had an HR of 1.57 (95%CI: 1.25–1.97, *P* = 0.001), and those that adopted cutoff method using other methods showed no statistically significant correlations. According to the median value of SUVmax, the groups of threshold were divided into two subgroups—high (≥5.55) and low (<5.55). Subgroup meta-analyses illustrated that the HRs of SUVmax were 1.20 (95% CI: 1.06–1.35, *P* = 0.07) and 2.34 (95% CI = 1.22–4.48, *P* = 0.0004) for high and low cut-off values. For analysis methods, the HRs of studies using univariate analysis was 2.01 (95%CI = 1.36–2.96, *P* = 0.55), and using multivariate analysis was 1.40 (95%CI = 1.13–1.73, *P* = 0.004). Based on the endpoint, eligible studies were divided into RFS group, DFS group and PFS group and EFS group, and subgroup analyses results showed that combined HR was 2.02(95% CI: 1.16–3.54, *P* = 0.005), 2.22(95% CI: 1.02–4.87, *P* = 0.12), 1.73(95% CI: 1.29–2.32, *P* = 0.24) and 1.09(95% CI: 1.01–1.17, *P* = 0.55).

**Table 4:** Subgroup of EFS and OS of SUV max.

Endpoint	Volumetric parameters	Factor	No. of studies	Heterogeneity test (I^2^, P)	Effect model	HR	95%CI of HR	Conclusion
EFS	SUV max	Cutoff method						
		ROC	11	65, 0.001	random	1.57	1.25,1.97	significant
		Others	3	42, 0.18	random	1.27	0.66,2.45	insignificant
		Threshold						
		≥5.55	9	44,0.07	fixed	1.20	1.06,1.35	significant
		<5.55	5	80,0.0004	random	2.34	1.22,4.48	significant
		Analysis method						
		Univariate analysis	5	0,0.55	fixed	2.01	1.36,2.96	significant
		Multivariate analysis	9	63,0.004	random	1.40	1.13,1.73	significant
		endpoint						
		RFS	5	73,0.005	random	2.02	1.16,3.54	significant
		DFS	2	58,0.12	random	2.21	0.66,7.42	significant
		PFS	5	28,0.24	fixed	1.73	1.29,2.32	significant
		EFS	2	0,0.55	fixed	1.09	1.01,1.17	significant
OS	SUV max	Cutoff method						
		ROC	7	66,0.008	random	1.70	1.07,2.69	significant
		Others	2	71,0.0006	random	1.01	0.96,1.06	insignificant
		Threshold						
		≥5.55	5	5,0.38	fixed	1.54	1.05,2.27	significant
		<5.55	4	85,0.0002	random	1.15	0.96,1.38	insignificant
		Analysis method						
		Univariate analysis	5	65,0.02	random	1.35	0.78,2.35	insignificant
		Multivariate analysis	4	63,0.04	random	1.67	0.96,2.89	insignificant

Abbreviations: HR = hazard ratios; CI = confidence interval, EFS = event-free survival; OS = overall survival; SUV max = maximum standard uptake value; MTV = metabolic tumor volume; TLG = total lesional glycolysis; RFS = recurrence/relapse free survival; PFS = progression-free survival; DFS = disease-free survival; ROC = receiver operating characteristic.

The EFS was based on 3 studies including MTV. A fixed-effects model was used and the pooled HR was 1.31(95% CI 0.65–2.65, *P* = 0.18; *I*^2^ = 42%, Fig 3). There was no significant heterogeneity, and so the results showed no statistically significant correlations.

The EFS was analyzed in 3 studies with TLG. Comprehensive data showed that higher TLG predicted lower EFS, and a fixed effects model (HR = 2.43; 95% CI = 1.28–4.64, *P* = 0.005; *I*^2^ = 81%) showed statistically significant heterogeneity (Fig 3), while random effects model (HR = 2.70; 95% CI = 0.54–13.44, P = 0.005) showed no statistically significant correlations. Sensitivity analysis was carried out to further estimate the impact of combined HRs, and the results revealed that heterogeneity was significantly reduced by the study of Yannan Zhao et al.(2018). [17] (*I*^2^ = 0%). The 95% CI of this sensitivity analysis was 2.57–13.71, *P* = 0.97, which indicated that TLG showed significant correlation with EFS. Potential publication bias was assessed by funnel plots (S2 Fig) and the results revealed no substantial asymmetry.

### Primary outcome: OS

The OS was based on 9 studies including SUVmax. According to the comprehensive data, the higher the SUVmax is, the worse was the OS according to the fixed effects model (HR = 1.06; 95% CI = 1.02–1.10, *P* = 0.006; *I*^2^ = 71%) and random effects model (HR = 1.22; 95% CI = 1.02–1.45, *P* = 0.006), (Fig 3). Potential publication bias was assessed by two statistical tests (Begg’s test and Egger’s test). Begg’s test (*P* = 1.000) and Egger’s test (*P* = 0.052), (S3 Fig) showed no significant publication bias. A sensitivity analysis was performed to further estimate the impact of combined HRs and omission of each study showed no significant reduction of heterogeneity.

Additional subgroup analyses were performed according to the cutoff method, threshold and analysis method (Table 4). Among studies that included OS, studies that adopted cutoff method using ROC had an HR of 1.70 (95%CI: 1.07–2.69, *P* = 0.008), and those that adopted cutoff method using other methods showed no statistically significant correlations. According to the median value of SUVmax, the groups of threshold were divided into two subgroups—high (≥5.55) and low (<5.55). Subgroup meta-analyses illustrated that the HRs of SUVmax had high cut-off values of 1.54 (95% CI: 1.05–2.27, *P* = 0.38); however, no statistically significant correlations were observed in the HRs with low cut-off values. For analysis methods, the HRs of studies using univariate analysis and multivariate analysis showed no significant results.

The OS was analyzed in 4 studies with MTV. Comprehensive data showed that OS prediction remained worse with higher MTV (HR = 2.91; 95% CI = 1.75–4.85, *P* = 0.44; *I*^2^ = 0%, (Fig 3), indicating that MTV was significantly correlated with OS. Potential publication bias was assessed by funnel plots (S4 Fig), and the results showed no substantial asymmetry.

The OS was analyzed in 3 studies with TLG, and fixed effects model was used (HR = 1.20; 95% CI = 0.65–2.23, P = 0.45; *I*^2^ = 0%, Fig 3). No significant heterogeneity was observed, and so the results showed no statistically significant correlations.

## Discussion

Clinicians often encounter the problem that many tumors, including breast cancer, are not effectively treated due to lack of standard treatment methods. Doctors and patients then need to reduce the side effects occurred due to failed treatments and avoid unnecessary treatments[31]. PET/CT technology is associated with high sensitivity (96%), strong specificity (94%), and is widely used in diagnosing and staging tumors, with 95% accuracy [32]. SUVmax, MTV and TLG are common parameters of PET/CT used in tumor diagnosis. These metabolic parameters also reflect the biological characteristics of tumors, thus providing some important information regarding clinical prognosis of tumor[33–36]. Patients might benefit if the values of these parameters help in predicting the EFS and OS of BC patients. According to Xia Q et al. (2015)[37], ^18^F- FDG/CT SUVmax value remained very effective in predicting the prognosis of liver metastasis of patients with colorectal cancer. A meta-analysis of 20 published studies was conducted to obtain evidence on the relationship of BC and SUVmax, MTV or TLG. Although SUVmax, MTV and TLG might be affected by varied reasons, our results indicated that patients with high SUVmax are at high risk of EFS along with poorer combined HRs [1.53(95% CI,1.25–1.89, *P* = 0.0006)] and patients with high TLG is associated with high risk of EFS along with poorer combined HRs [5.94(95% CI, 2.57–13.71, *P* = 0.97)], while SUVmax and MTV is associated with high risk of OS in patients along with poorer combined HRs [1.22 (95% CI, 1.02–1.45, *P* = 0.0006)] and [2.91(95% CI, 1.75–4.85, *P* = 0.44)]. Our meta-analysis results did not reveal the prognostic value of MTV for EFS [HR = 1.31(95% CI 0.65–2.65, *P* = 0.18), Fig 3] and TLG for OS[HR = 1.20 (95% CI = 0.65–2.23, *P* = 0.45), Fig 3], as they are influenced by limited sample size, which in turn result in low statistical efficiency. This might be affected by insufficient statistical power, since there were only 3 studies analyzed EFS with MTV and 3 studies analyzed EFS with TLG. Further research should be conducted to investigate the prognostic value of MTV for EFS and TLG for OS in patients with BC.^18^ F-FDG-PET/CT can be used for risk stratification in disease control and survival. Future large-scale prospective studies are warranted to further validate our findings.

Significant heterogeneity was found for SUVmax in predicting EFS and OS. According to the guidelines and protocols for ^18^ F-FDG PET imaging, heterogeneity of PET/CT parameters (duration of fasting, preinjection blood glucose test, post-injection interval, and dose of ^18^F-FDG) included in this study were acceptable as the values were within normal range [3, 38, 39](Table 2). To investigate the source of heterogeneity, cutoff method, threshold, and analysis method were used to conduct subgroup analysis of both EFS and OS, and EFS was used as an endpoint to conduct subgroup analysis (Table 3). Firstly, cutoff method was used to divide the data into two subgroups, in which the ROC group showed no significantly reduced heterogeneity, while others showed no statistically significant correlations. Secondly, the optimal cut-off value of each study was different, and so we divided the study into two groups, with the median value of 5.55. The subgroup with threshold above 5.55 is considered homogeneous *(I* ^2^ = 44%, *P* = 0.007). Thirdly, multivariate and univariate methods were adopted to extract HR to study the heterogeneity source of HR. The results showed that heterogeneity between univariate groups was significantly reduced (*I*^2^ = 0, *P* = 0.55). In the four subgroups with different survival endpoints, only two subgroups, PFS (*I*^2^ = 28, *P* = 0.24) and EFS (*I*^2^ = 0, *P* = 0.55), showed significant heterogeneity reduction. So, threshold, source of HR and endpoint are considered to be sources of heterogeneity on EFS. Similarly, cutoff method, threshold, analysis method were used to conduct subgroup analysis on OS. The results showed no reduction in heterogeneity in the subgroups using cutoff method or analysis method. Interestingly, the subgroup with threshold above 5.55 was considered to be homogeneous (*I* ^2^ = 5%, *P* = 0.38). Then threshold was regarded as a source of heterogeneity for OS. Our subgroup analysis showed that SUVmax as a significant risk factor for both EFS and OS in BC patients with SUVmax above median value of 5.55, and it is a reason of great satisfaction for us. But we were unable to determine an optimal cut-off value to SUVmax. Different cut-off values and delineation strategies, and various histological methods were used in the studies, which might affect the occurrence of events as well as survival. Further studies with data from individual patients are needed to determine the standard cut-off values and delineation methods for predicting the prognosis using SUVmax.

Significant heterogeneity was also found for TLG in predicting EFS. Three studies confirmed the relationship between TLG and EFS, and fixed effects model showed significant correlation of TLG with EFS. Sensitivity analysis found that the study conducted by Yannan Zhao et al.(2018)[17] was the cause of heterogeneity. Random effects model revealed substantial changes (HR = 2.70; 95% CI = 0.54–13.44) and showed no statistically significant correlation with EFS. Yannan Zhao et al. (2018) used fulvestrant to treat BC, but it is not a regular BC treatment drug. No similar study was found in our research. Several larger sample size studies are needed in future to find out whether fulvestrant has an impact on EFS in BC patients with high TLG. After discussion, we considered that this study would not be included as prognosis of EFS in BC patients has high TLG for time being.

Moreover, the quality of included studies should also be taken into account as it is a limitation of our study. Firstly, although all the included studies were evaluated by Cochrane risk bias tool and included high quality studies, some studies still lacked partial details of patient and data of ^18^ F-FDG PET scan. Furthermore, prospective studies combining survival rate of BC and PET parameters are needed. Secondly, BC is a heterogeneous disease, and patients with different histological grades, stages, and treatments were included in this meta-analysis, which can affect the events occurring over time and survival. Thirdly, as far as we know, there are some studies on PET parameters of tumors or lymph nodes, but our study focused only on tumor parameters. Fourthly, non-English articles were excluded in this study, which might lead to potential impact of language bias. Fifthly, only published studies were included when searching the electronic databases, and so publication bias cannot be excluded. However, evaluation of publication bias suggested that our analysis was reliable. Sixthly, Engauge Digitizer was used to extract the data of HRs from survival curves indirectly, leading to an imprecision. Finally, studies included in this meta-analysis are almost conducted in Asia, and the incident of BC is high in these regions and race of humans in these countries might cause bias.

## Conclusion

Despite the adoption of different methods for different types of BC patients, the present meta-analysis confirmed that BC patients with high SUVmax are at high risk of adverse events or even death, while MTV is associated with high risk of death and TLG is associated with high risk of adverse events. However, our meta-analysis did not reveal the prognostic value of MTV for adverse events and TLG for death.

## Supporting information

S1 Fig Egger’s test of SUV max and EFS. (EPS)

S2 Fig Funnel plots of EFS and TLG. (EPS)

S3 Fig Egger’s test of SUV max and OS. (EPS)

S4 Fig Funnel plots of MTV and OS. (EPS)

S1 File PRISMA checklist. (DOC)
