# Supplementary material for: Prognostic value of maximum standard uptake value, metabolic tumor volume, and total lesion glycolysis of positron emission tomography/computed tomography in patients with breast cancer: A systematic review and meta-analysis
Source: PLoS One. 2019 Dec 11;14(12):e0225959. doi: 10.1371/journal.pone.0225959 (PMC6905566; doi:10.1371/journal.pone.0225959)
Supplement: S1 File — (DOC) [file pone.0225959.s005.doc]

| **Section/topic** | **#** | **Checklist item** | **Reported on page #** |
| --- | --- | --- | --- |
| **TITLE** | | |  |
| Title | 1 | **Prognostic value of maximum standard uptake value,** **metabolic tumor volume, and total lesion glycolysis of positron emission tomography/computed tomography in patients with breast cancer: A systematic review and meta-analysis** | 1 |
| **ABSTRACT** | | |  |
| Structured summary | 2 | **Purpose:** A comprehensive systematic review of the literature was conducted on parameters from ^18^ F-FDG PE and a meta-analysis of the prognostic value of the maximal standard uptake value (SUVmax), metabolic tumor volume (MTV) and total lesional glycolysis (TLG) in patients with breast cancer (BC).  **Patients and methods:** Relevant English articles from PubMed, EMBASE, and the Cochrane Library were retrieved. Pooled hazard ratios (HRs) were used to assess the prognostic value of SUVmax, MTV, and TLG.  **Results:** A total of 20 primary studies with 3115 patients with BC were included. The combined HRs (95% confidence interval [CI] of higher SUVmax and higher TLG for event-free survival (EFS) were 1.53 (95% CI, 1.25-1.89, *P*=0.0006) and 5.94 (95% CI, 2.57-13.71, *P* =0.97), respectively. Regarding the overall survival (OS), the combined HRs were 1.22 (95%CI, 1.02-1.45, *P*=0.0006) with higher SUVmax, and 2.91(95% CI, 1.75-4.85, *P*=0.44) with higher MTV. Higher MTV showed no correlation with EFS [1.31(95% CI, 0.65-2.65, *P*=0.18)] and similarly higher TLG showed no correlation with OS [1.20(95% CI, 0.65-2.23, *P*=0.45)]. [Subgroup](javascript:;) [analysis](javascript:;) showed that SUVmax, with a median value of 5.55 was considered as a significant risk factor for both EFS and OS in BC patients.  **Conclusion:** Despite clinically heterogeneous BC patients and adoption of various methods between studies, the present meta-analysis results confirmed that patients with high SUVmax are at high risk of adverse events or death in BC patients,  high MTV predicted a high risk of death and high TLG predicted a high risk of adverse events.  **Keywords:** SUVmax, MTV, TLG, breast cancer, positron emission tomography/computed tomography, meta-analysis | 2 |
| **INTRODUCTION** | | |  |
| Rationale | 3 | Breast cancer (BC) is the most common malignancy in women. Although new imaging tools and assisted systemic therapy have improved the survival rate of patients with BC, patients with early invasive BC are still at risk of recurrence or death. It is crucial to identify patients experiencing a risk of relapse or progression, as there is no clinical method for accurate assessment of the prognosis and survival of BC patients till date.According to the latest report, tumor size, nuclear grade, axillary lymph node involvement, hormone receptor (e.g., estrogen receptor (ER) progesterone) status receptor (PR) and human epidermal growth factor receptor 2 (HER2), and ki-67 proliferation index might act as effective factors in predicting the recurrence or progression in patients at high risk. A growing body of evidence suggests that fluoro18-fluorodeoxyglucose (^18^F- FDG) positron emission tomography (PET/CT) has a great prognostic significance in predicting malignant tumors, TNM staging, evaluation of therapeutic effects, FDG parameter SUV Max, metabolic tumor volume (MTV) and total lesional glycolysis (TLG), as a parameter of tumor metabolism and volume have also received more and more attention. MTV is the size of the tumor tissue, which actively ingests ^18^F- FDG, and TLG is the median SUV value in the region of interest MTV . | 3 |
| Objectives | 4 | However, it is still controversial whether the parameters of ^18^F-FDG PET/CT predict the survival rate of BC patients. Some studies reported significant relationships between high SUV max and poor prognoses in patients with BC, whereas no such correlation is observed by Alexandre Cochet et al. **.** Therefore, a meta-analysis was designed to evaluate the prognostic value of SUV max MTV and TLG in BC patients. | 3 |
| **METHODS** | | |  |
| Protocol and registration | 5 | This systematic review and meta-analysis was reported by following the guidelines of preferred reporting items of the systematic review and meta-analysis (PRISMA) statement. | 4 |
| Eligibility criteria | 6 | Inclusion criteria were as follows: (1) studies should include histologically diagnosed BC patients; (2) 18F-FDG PET/CT was used as imaging tool before treatment; (3) the study should at least report one form of survival data; and (4) articles published in English. | 4 |
| Information sources | 7 | A systematic search of PubMed, Embase, and Cochrane Library (2012-May 2019) | 4 |
| Search | 8 | Use the following keywords (“breast cancer” OR “breast carcinoma”)AND(“positron emission tomography” OR “positron emission tomography-computed tomography” OR “positron emission tomography computed tomography” OR “PET”OR “PET-CT” OR “PET CT” OR “PET/CT” OR“fluorodeoxyglucose” OR“FDG”) AND (“prognostic” OR “prognosis” OR “predictive” OR “survival” OR “outcome”) | 4 |
| Study selection | 9 | Inclusion criteria were as follows: (1) studies should include histologically diagnosed BC patients; (2) 18F-FDG PET/CT was used as imaging tool before treatment; (3) the study should at least report one form of survival data; and (4) articles published in English. The exclusion criteria were as follows: (1) studies that focused only on diagnosis, staging, or monitoring recurrence or progression; (2) studies involving patients with recurrent or distant metastatic disease before treatment; and (3) reviews, case reports, conference abstracts and editorial materials. two authors independently conducted the search and screening, and any discrepancies were resolved by reaching a consensus. If the results reported are from the same sample, completed studies with the latest information will be used. | 4 |
| Data collection process | 10 | Two authors (W Wen and D Xu) independently extracted the following data regarding the included studies (Table 1): (1) basic information of the study, including the year of publication, first author, study time, follow-up duration and study design; (2) details of patients and tumors, including median age, sample size, histology, TNM staging, treatment measures and endpoint. The information regarding 18F-FDG- PET scan data and parameters, determination of fasting time before injection, blood glucose detection before injection, determination of truncated interval value of FDG injection dose, extraction of truncated value of PET parameters SUV Max, MTV, TLG, and tumor profile was also extracted and presented in Table 2. | 4-8 |
| Data items | 11 | We followed the same methodology as used in our previous study. Event-free survival (EFS) is defined as the time from treatment initiation to recurrence or progression. In this meta-analysis, disease-free survival (DFS), progression-free survival (PFS), and disease-free metastasis survival in the included studies were combined and redefined as EFS. Overall survival (OS) was defined as the time from therapy initiation till death regardless of the causes. As the effect size of each study, hazard ratio (HR) and 95% confidence interval (CI) take into account the number and time of events. Measure the effect of 18F-FDG PET parameters on survival outcome through effect size of HR in order to measure the correlation between SUV max, MTV and TLG values and the prognosis of BC patients. | 13 |
| Risk of bias in individual studies | 12 | The quality of 20 studies was assessed using the Cochrane risk of bias tool (figure 2), in which few studies were non-blinded or non-randomized. In our study, follow-up data from 6 studies were missing. Most of the studies were well described and monitored regarding adverse events by objective criteria, although some studies involve different types of study designs and definitions. | 9 |
| Summary measures | 13 | hazard ratio (HR) and 95% confidence interval (CI) take into account the number and time of events. | 9 |
| Synthesis of results | 14 | Statistical heterogeneity was measured using chi-squared Q test and I^2^ statistic. Heterogeneity was considered to be present if *P*<0.05 or/and *I* ^2^ >50%. A fixed effects model was used for meta-analysis when heterogeneity was not significant, while a random effects model was used if heterogeneity was significant. RevMan version 5.3 (RevMan, version 5.3; The Nordic Cochrane Centre, The Cochrane Collaboration) and STATA version 12.0 (STATA Corp., College Station, TX) were used for statistical analysis. | 10 |

Page 1 of 2

| **Section/topic** | **#** | **Checklist item** | **Reported on page #** |
| --- | --- | --- | --- |
| Risk of bias across studies | 15 | Begg’s test and Egger’s test were used for evaluating bias by STATA version 12.0. P values of less than 0.05 were considered to be statistically significant. | 10 |
| Additional analyses | 16 | Additional subgroup analyses were performed according to the cutoff method, threshold, analysis method and endpoint. | 12 |
| **RESULTS** | | |  |
| Study selection | 17 | The search process of the literature was presented in figure 1. Search was conducted in three databases, which obtained 559 Embase articles, 1149 PubMed articles and 20 Cochrane Library articles initially (1728 articles). After excluding duplications and meeting summaries, 69 articles that did not meet the inclusion criteria were removed. Finally, 20 studies including 3,115 patients that met the conditions of the study and published from 2013 to 2019 were included in this meta-analysis (figure 1). | 10 |
| Study characteristics | 18 | Almost all the studies were conducted in Asia, with 9 in South Korea, 4 in China and 2 in Japan, 3 in the United States and 1 each in France and Spain. Two were prospective and 18 were retrospective studies. In the 18 SUVmax studies, the SUV cut-off values ranged from 2.5-11.1, which included 14 items with EFS as prognosis and 9 items with OS as prognosis. Among the 6 studies that measured MTV, 3 included EFS as prognosis and 4 included OS as prognosis. Among the 6 studies measuring TLG, 3 with EFS as prognosis and 3 with OS as prognosis were included. In addition, information such as age of the subjects during the follow-up period of tumor pathological staging was extracted. The details of all studies included in the analysis, histology and treatment included in all studies are presented in table 1. Sixty-five percent of patients are with invasive ductal carcinoma (IDC), invasive lobular carcinoma (ILC), and other pathologies. One study involved patients with inflammatory BC, and three studies included only patients with advanced metastatic BC and all these included one or more treatments of surgery (SG)/chemotherapy (CMT)/radiotherapy (RT)/ endocrine therapy (ET)/ hormone /neoadjuvant chemotherapy (NAC). Yannan Zhao et al. 's study involves experimental treatment of metastatic BC with fulvestrant. | 11 |
| Risk of bias within studies | 19 | Fixed effects model (HR =1.14; 95% CI = 1.07-1.21, *P*=0.0006; *I^2^* = 64%) showed statistical significance, and heterogeneity existed between studies, while random effects model [HR =1.53; 95% CI = 1.25-1.89, *P*=0.0006 (figure 3A)] still showed meaningful results (Table 3). Potential publication bias was assessed by two statistical tests (Begg’s and Egger’s). Begg’s test showed no significant publication bias (*P*=0.352), and Egger’s test (Supplement Figure 1) indicated that there might be publication bias (*P*=0.002). Therefore, trim and fill analysis was conducted to ensure the reliability of combined HR. Symmetrical funnel plots were obtained after trim and fill analysis (figure 4). After adding the hypothesis literature, the results were obtained (HR = 1.104; 95% CI : 1.040-1.172), and no substantial change was observed in the results before and after adding the hypothesized literatures , which still showed that SUVmax was significantly correlated with EFS. | 11-12 |
| Results of individual studies | 20 | Fourteen studies analyzed EFS with SUVmax. After combining HR, the higher SUVmax, and the worse EFS are predicted. Fixed effects model (HR =1.14; 95% CI = 1.07-1.21, *P*=0.0006; *I^2^* = 64%) showed statistical significance, and heterogeneity existed between studies, while random effects model [HR =1.53; 95% CI = 1.25-1.89, *P*=0.0006 (figure 3A)]  The EFS was based on 3 studies including MTV. A fixed-effects model was used and the pooled HR was 1.31(95% CI 0.65-2.65, *P*=0.18; *I*^2^ = 42%, figure 3B). | 11 |
| Synthesis of results | 21 | Fourteen studies analyzed EFS with SUVmax. random effects model [HR =1.53; 95% CI = 1.25-1.89, *P*=0.0006 (figure 3A)] still showed meaningful results (Table 3) | 11 |
| Risk of bias across studies | 22 | Potential publication bias was assessed by two statistical tests (Begg’s and Egger’s). Begg’s test showed no significant publication bias (*P*=0.352), and Egger’s test (Supplement Figure 1) indicated that there might be publication bias (*P*=0.002). | 11 |
| Additional analysis | 23 | Additional subgroup analyses were performed according to the cutoff method, threshold, analysis method and endpoint (Table4). Among the studies that included EFS as endpoint, studies that adopted cutoff method using ROC had an HR of 1.57 (95%CI: 1.25-1.97, *P* = 0.001), and those that adopted cutoff method using other methods showed no statistically significant correlations. According to the median value of SUVmax, the groups of threshold were divided into two subgroups—high (≥5.55) and low (<5.55). Subgroup meta-analyses illustrated that the HRs of SUVmax were 1.20 (95% CI: 1.06-1.35, *P* = 0.07) and 2.34 (95% CI = 1.22-4.48, *P* = 0.0004) for high and low cut-off values. For analysis methods, the HRs of studies using univariate analysis was 2.01 (95%CI = 1.36-2.96, *P* = 0.55), and using multivariate analysis was 1.40 (95%CI = 1.13-1.73, *P* = 0.004). Based on the endpoint, eligible studies were divided into RFS group, DFS group and PFS group and EFS group, and subgroup analyses results showed that combined HR was 2.02(95% CI: 1.16-3.54, *P* = 0.005), 2.22(95% CI: 1.02-4.87, *P* = 0.12), 1.73(95% CI: 1.29-2.32, *P* = 0.24) and 1.09(95% CI: 1.01-1.17, *P* = 0.55). | 12-13 |
| **DISCUSSION** | | |  |
| Summary of evidence | 24 | A meta-analysis of 20 published studies was conducted to obtain evidence on the relationship of BC and SUVmax, MTV or TLG. Although SUVmax, MTV and TLG might be affected by varied reasons, our results indicated that patients with high SUVmax are at high risk of EFS along with poorer combined HRs [1.53(95% CI,1.25-1.89, *P*=0.0006)] and patients with high TLG is associated with high risk of EFS along with poorer combined HRs [5.94(95% CI ,2.57-13.71, *P*=0.97)], while SUVmax and MTV is associated with high risk of OS in patients along with poorer combined HRs [1.22 (95% CI , 1.02-1.45, *P*=0.0006)] and [2.91(95% CI ,1.75-4.85, *P*=0.44)]. Our meta-analysis results did not reveal the prognostic value of MTV for EFS and TLG for OS, as they are influenced by limited sample size, which in turn result in low statistical efficiency. ^18^ F-FDG-PET/CT can be used for risk stratification in disease control and survival. | 16 |
| Limitations | 25 | Firstly, although all the included studies were evaluated by Cochrane risk bias tool and included high quality studies, some studies still lacked partial details of patient and data of ^18^ F-FDG PET scan. Furthermore, prospective studies combining survival rate of BC and PET parameters are needed. Secondly, BC is a heterogeneous disease, and patients with different histological grades, stages, and treatments were included in this meta-analysis, which can affect the events occurring over time and survival. Thirdly, as far as we know, there are some studies on PET parameters of tumors or lymph nodes, but our study focused only on tumor parameters. Fourthly, non-English articles were excluded in this study, which might lead to potential impact of language bias. Fifthly, only published studies were included when searching the electronic databases, and so publication bias cannot be excluded. However, evaluation of publication bias suggested that our analysis was reliable. Sixthly, Engauge Digitizer was used to extract the data of HRs from survival curves indirectly, leading to an imprecision. Finally, studies included in this meta-analysis are almost conducted in Asia, and the incident of BC is high in these regions and race of humans in these countries might cause bias. | 18 |
| Conclusions | 26 | Despite the adoption of different methods for different types of BC patients, the present meta-analysis confirmed that BC patients with high SUVmax are at high risk of adverse events or even death, while MTV is associated with high risk of death and TLG is associated with high risk of adverse events. However, our meta-analysis did not reveal the prognostic value of MTV for adverse events and TLG for death. | 19 |
| **FUNDING** | | |  |
| Funding | 27 | This research was supported by the National Natural Science Foundation of China (31760330) and (81560400). | 19 |

*From:*  Moher D, Liberati A, Tetzlaff J, Altman DG, The PRISMA Group (2009). Preferred Reporting Items for Systematic Reviews and Meta-Analyses: The PRISMA Statement. PLoS Med 6(7): e1000097. doi:10.1371/journal.pmed1000097

For more information, visit: **www.prisma-statement.org**.

Page 2 of 2
