# Supplementary material for: Prognostic value of maximum standard uptake value, metabolic tumor volume, and total lesion glycolysis of positron emission tomography/computed tomography in patients with breast cancer: A systematic review and meta-analysis
Source: PLoS One. 2019 Dec 11;14(12):e0225959. doi: 10.1371/journal.pone.0225959 (PMC6905566; doi:10.1371/journal.pone.0225959)
Supplement: Attachment — Submitted filename: Response to Reviewers.docx [file pone.0225959.s006.docx]

**Response to** Reviewers

**Point by point reply to the comments**

Answer to Reviewer #1:

1. I understand that MTV and TLG have not a significant correlation with PFS and OS in the meta-analysis, but authors did not clearly explain this data in the results and discussion section

Answer: Thank you for your valuable advice. I have supplemented the data in the results section and the discussion section according to your requirements, It is also marked in red in the original text.

In the results section: The EFS was based on 3 studies including MTV. A fixed-effects model was used and the pooled HR was 1.31(95% CI 0.65-2.65, *P*=0.18; *I*^2^ = 42%, figure 3B). There was no significant heterogeneity, and so the results showed no statistically significant correlations. The OS was analyzed in 3 studies with TLG, and fixed effects model was used (HR =1.20; 95% CI = 0.65-2.23, P=0.45; *I*^2^ = 0%, figure 3F). No significant heterogeneity was observed, and so the results showed no statistically significant correlations.

In the discussion section: Our meta-analysis results did not reveal the prognostic value of MTV for EFS [HR=1.31(95% CI 0.65-2.65, *P*=0.18) , figure 3B] and TLG for OS[HR=1.20 (95% CI = 0.65-2.23, *P*=0.45), figure 3F].

2. Only SUVmax significantly correlate with PFS and OS, so authors should rewrite the paper according to this main significant result.

Answer: Thank you for your valuable advice. I have rewritten the discussion of MTV for EFS and TLG for OS according to your requirements. Additional discussion sections are as follows: Our meta-analysis results did not reveal the prognostic value of MTV for EFS [HR=1.31(95% CI 0.65-2.65, *P*=0.18) , figure 3B] and TLG for OS[HR=1.20 (95% CI = 0.65-2.23, *P*=0.45), figure 3F], as they are influenced by limited sample size, which in turn result in low statistical efficiency. This might be affected by insufficient statistical power, since there were only 3 studies analyzed EFS with MTV and 3 studies analyzed EFS with TLG. Further research should be conducted to investigate the prognostic value of MTV for EFS and TLG for OS in patients with BC.^18^ F-FDG-PET/CT can be used for risk stratification in disease control and survival. Future large-scale prospective studies are warranted to further validate our findings.

Answer to Reviewer #2:

Thanks for your valuable comments on my paper. I have benefited a lot. The following is the answer to your question: The quality assessment is not appropriate. Actually the authors used the quality assessment tools for treatment studies. For prognostic studies, there are couple of quality assessment tools for example the following link can be used
<https://www.cebm.net/wp-content/uploads/2018/11/Prognosis.pdf>

Answer: The following is what I modified and added according to your requirements and it is also marked in red in the original text. We also modified the quality evaluation chart. The rewrite is as follows: The quality of 20 studies was assessed according to CRITICAL APPRAISAL OF PROGNOSTICSTUDIES

(<https://www.cebm.net/wp-content/uploads/2018/11/Prognosis.pdf)(figure>2). Generally, the included studies were of [high](javascript:;) [quality](javascript:;)，In the domain of prognostic factor follow-up time measurements , there was a high risk of bias in 7 studies because follow-up data were missing Or the follow-up time was too short. 7 studies were judged to be at high or unclear risk of bias in the domain of defined representative sample measurements because few studies were non-blinded or non-randomized. Most of the studies were well described and monitored regarding adverse events by objective criteria.
